# Exploring the Bidirectional Relationship Between Numerical Cognition and Motor Performance: A Systematic Review

**DOI:** 10.3390/brainsci15121331

**Published:** 2025-12-14

**Authors:** Eliane Rached, Jihan Allaw, Joy Khayat, Hassan Karaki, Ahmad Diab, Antonio Pinti, Ahmad Rifai Sarraj

**Affiliations:** 1 Laboratory of Motor System, Handicap and Rehabilitation (MOHAR), Faculty of Public Health, Lebanese University, Beirut P.O. Box 6573/14, Lebanon; eliane.rached.1@ul.edu.lb (E.R.); jihan.allaw.1@ul.edu.lb (J.A.); joykhayat@hotmail.com (J.K.); hkaraki@ul.edu.lb (H.K.); 2 Laboratoire de Recherche Sociétés et Humanité (LARSH DeVisu), Université Polytechnique Hauts-De-France, 59300 Valenciennes, France; antonio.pinti@uphf.fr; 3 Signal Processing Research Group, Biomedical Engineering Department, Lebanese International University, Tripoli P.O. Box 146404, Lebanon; ahmad.diab@liu.edu.lb

**Keywords:** numerical cognition, embodied cognition, arithmetic, mental calculation, motor performance, motor function

## Abstract

Background: Numerical cognition and motor performance rely on overlapping brain systems, yet the extent of their reciprocal interaction remains unclear. This systematic review explores how number processing influences motor execution and how motor activity shapes numerical cognition, emphasizing the neural mechanisms underlying these associations. Methods: A comprehensive search of Scopus, PubMed, MEDLINE, SPORTDiscus, PsycINFO, and SpringerLink, as well as journal citations and conference proceedings (up to August 2025), identified experimental studies examining the interplay between numerical cognition and motor performance in healthy adults. Both randomized and non-randomized designs were included. Two reviewers independently screened, extracted data, and assessed study quality following PRISMA and Cochrane Risk of Bias guidelines. Results: Twelve studies met the inclusion criteria. Most showed that numerical stimuli facilitated motor responses, with congruent number–movement pairings yielding faster reactions and more efficient kinematics. Mental calculation often enhanced motor output (e.g., force, jump height), though interferences emerged under high cognitive load. Conversely, motor actions consistently biased numerical judgments, aligning with spatial–numerical associations. Conclusions: Evidence suggests a predominant pattern of facilitation, likely reflecting shared networks between cognitive and motor resources. These findings advance theoretical understanding and highlight promising translational applications in education, sport, and neurorehabilitation.

## 1. Introduction

Numerical cognition—the ability to understand, represent, and manipulate numerical information—encompasses multiple components, including basic number sense, magnitude comparison, symbolic representation, and arithmetic operations [1].

Recent cognitive neuroscience and developmental research increasingly emphasize the interdependence of numerical cognition and motor functions, challenging traditional views that consider these domains as largely distinct. Instead, perception, action, and higher-order cognition are now understood as deeply intertwined through shared representational formats and overlapping neural mechanisms [2]. This perspective is supported by growing evidence that numerical cognition is profoundly embodied, involving motor systems alongside conventional number-processing brain regions [3].

Neuroanatomically, numerical cognition consistently engages distributed fronto-parietal and prefrontal networks, including the intraparietal sulcus (IPS), angular gyrus, and dorsolateral prefrontal cortex [4,5]. These regions, central to numerical processing, also subserve visuospatial attention, motor preparation, and execution, implying that numerical and motor domains share critical neural substrates.

This shared neural architecture appears to be evolutionarily ancient. Nieder (2016) identified ‘number neurons’ within the intraparietal sulcus and prefrontal cortex of macaques that are selectively tuned to numerosity [6]. These neurons exhibit tuning curves centered on preferred numerosities and adhere to Weber–Fechner scaling, characteristic of approximate magnitude encoding. Crucially, these neurons are present in animals lacking symbolic number training, suggesting that numerosity is encoded as an abstract stimulus property rather than merely through learned sensorimotor associations. Because these number-selective neurons reside within fronto-parietal circuits essential for motor planning, they provide a neuroanatomical basis for numerical magnitude to directly influence motor parameters—such as force and trajectory—prior to the engagement of learned routines. This finding extends the concept of a ‘number sense’ to non-human animals and underscores the necessity of distinguishing between effects driven by intrinsic core magnitude representations and those modulated by well-established motor experience. Consequently, novel, minimally practiced tasks may offer a clearer window into these intrinsic magnitude–motor mappings compared to overlearned actions, where automatic motor patterns may confound the results.

A seminal theoretical framework in this field is the Triple Code Model (TCM) [4,5], which proposes three distinct representational codes for numerical information: (1) a visual Arabic code localized primarily in occipito-parietal areas; (2) a verbal code associated with left perisylvian and angular gyrus regions; and (3) an analog magnitude code situated bilaterally in the intraparietal sulcus (IPS), encoding approximate quantity. Significantly, the IPS also supports sensorimotor transformations and spatial coding, establishing it as a neural connection mediating interactions between numerical cognition and motor control.

Neuroimaging studies reveal that cerebral regions commonly associated with motor control, such as the premotor cortex and supplementary motor area, are mobilized during numerical tasks [7,8]. Also, the intraparietal sulcus, a core for numerical magnitude processing, operates alongside these motor areas, suggesting a distributed network that integrates numerical and motor functions [9]. For instance, finger counting habits modulate motor cortex activation during numerical processing, indicating that motor areas contribute substantively to numerical understanding rather than serving as incidental correlates [10]. Also, recent neuroimaging investigations further underscore the parietal cortex’s pivotal role in the dynamic integration of numerical and motor representations during task performance [11,12].

Complementing the TCM, Walsh’s A Theory of Magnitude (ATOM) [13] offers a unifying framework, proposing that time, space, number, and action are processed within a shared cortical substrate predominantly localized in the parietal cortex. This framework suggests that abstract cognitive operations such as quantity judgment and arithmetic, alongside motor functions like spatial planning and force scaling, rely on an underpinning common magnitude-processing networks. Empirical evidence has increasingly supported ATOM’s predictions, demonstrating coordinated activity in fronto-parietal and motor-related regions during tasks that demand simultaneous numerical and motor processing [14].

From a motor control perspective, the execution and regulation of movement involve coordinated activity across the primary motor cortex, premotor cortex, supplementary motor area, basal ganglia, cerebellum, and parietal regions [15,16]. These structures facilitate not only precise voluntary movements but also higher-order functions including action selection, predictive control, and sensorimotor integration. Notably, the parietal cortex integrates visuospatial information essential for both motor planning and numerical representation, while the cerebellum and basal ganglia contribute to timing, sequencing, and automatization across motor and cognitive domains. Recent studies highlight the engagement of sensorimotor circuits in numerical magnitude processing and response preparation [17], further bridging motor and cognitive neuroscience.

This convergence of evidence supports embodied cognition theories, which argue that abstract numerical concepts are anchored in sensorimotor experiences rather than just existing as purely abstract mental entities [3] meaning it is grounded in perceptual and motor systems [18,19]. Phenomena such as the Spatial-Numerical Association of Response Codes (SNARC) effect exemplifies this grounding, revealing systematic associations between number magnitude and spatial-motor responses, with smaller numbers linked to leftward and larger numbers to rightward actions [20,21], reflecting a spatially organized mental number line closely tied to motor output. Moreover, behavioral and neurophysiological correlates including variations in grip aperture, saccadic eye movements, and manual reaching scaled by numerical magnitude, provide convergent evidence that numeric representations directly influence sensorimotor processes [22]. Recent meta-analytic findings confirm a medium effect size of embodied cognition on number processing, emphasizing the sensorimotor foundation of numerical representations [23].

Developmental research complements these neural findings, demonstrating that fine motor skills and finger gnosis—the ability to perceive and control one’s fingers—predict stronger numerical and arithmetic skills in children [10]. Impairments in fine motor control correlate with delays in numerical development, suggesting that sensorimotor experiences such as finger counting and manipulation facilitate the acquisition of symbolic number knowledge [24]. This link has been documented across cultures and languages [25], highlighting the fundamental role of sensorimotor systems in numerical cognition [26].

To sum up, the evidence from neuroimaging and developmental studies reveals a complex and bidirectional interaction between motor functions and numerical cognition. The involvement of motor regions such as the premotor cortex and the importance of finger gnosis underscore that numerical understanding is not solely a mental abstraction but is deeply rooted in bodily experiences and sensorimotor pathways [27]. In addition, the influence of numerical cognition on motor performance is equally remarkable, with numerical tasks modulating hand movements, response speed, and even postural control [28], particularly during spatially and sequentially demanding operations like subtraction or mental number line estimation [29,30].

Despite growing interest, the reciprocal relationship between numerical cognition and motor performance remains underexplored in a comprehensive and systematic manner. Most extant studies focus on unidirectional effects, such as how movement alters numerical processing, often without controlling movement parameters, such in the case of gait trajectory, fingers, hand, or even upper limb movements, performed without constraints as to the intensity of movement. To our knowledge, no prior systematic review has comprehensively synthesized evidence on the reciprocal relationship between numerical cognition and motor performance. By integrating the limited but growing literature, this review would establish a foundational framework for future empirical investigations and practical applications. Moreover, although behavioral findings are relatively well documented, the neural mechanisms underpinning this interaction have received limited attention, leaving critical gaps regarding the specific brain networks mediating cognitive-motor coupling.

This review aims to address these gaps by: (1) synthesizing empirical findings on the reciprocal interactions between numerical cognition and motor performance, investigating how numerical tasks engagement (e.g., arithmetic, magnitude comparison) influences motor execution and coordination, and how motor experiences and interventions (e.g., finger training, whole-body movement, action-based learning) impact numerical cognition development and enhancement; (2) assessing whether these interactions predominantly facilitate or interfere with task execution; and (3) situating these behavioral effects within a cognitive neuroscience framework, emphasizing the involvement of fronto-parietal, premotor, cerebellar, and basal ganglia circuits.

By bridging behavioral data with theoretical models and neuroanatomical evidence, this review seeks to deepen our understanding of the intricate neural and cognitive connections between numerical processing and motor function, highlighting promising avenues for translational applications in education, sports science, and neurorehabilitation.

## 2. Methods

### 2.1. Criteria for Considering Studies for This Review

#### 2.1.1. Types of Studies

We included randomized and non-randomized controlled trials (RCTs and non-RCTs) besides observational studies, examining the relationship between arithmetic and motor actions. Both randomized and non-randomized experimental studies were included due to the exploratory and emerging nature of research on number–motor interactions. Given the limited number of available studies, exclusion based solely on randomization was not applied. Study quality was instead furtherly addressed through risk-of-bias assessment using ROBINS-I. Included studies had to be written in English.

#### 2.1.2. Types of Participants

Trials were included when all the participants were novice, adults or older adults. Even though older adults are considered 65 years and older and more prone to morbidities and comorbidities, this step was done in order to avoid the exclusion of relevant trials, though under specified and restricted health criteria.

Consequently, excluded trials were the one that recruited (a) children or adolescents as population; (b) athletes or sports amateurs; (c) cognitive impairments or neurological and psychological diseases, and (d) participants with a history of orthopedic surgeries in both upper or lower limbs and spine.

#### 2.1.3. Types of Interventions

Included trials were the ones that (a) compare the use of a numerical cognitive strategy (e.g., mental arithmetic, number comparison, number line estimation) in relation to motor activity or against a control condition, (b) compare any motor action (e.g., reaching task, walking, postural control, finger tapping) in relation to numerical cognition tasks or against a control condition, (c) investigates a pre-post intervention effect related to stated conditions above; (d) for studies conducting the effect of arithmetic on motor performance, they had to measure motor performance, motor function, motor action and their variants as a dependent variable; (e) for studies investigating the effect of motor performance on numerical cognition, they had to measure arithmetical task or number processing capacity as a dependent variable.

Trials focusing on dual task intervention were excluded since they do not align with the objective of this review.

#### 2.1.4. Types of Outcome Measures

Trials assessing numerical cognition, motor performance and related constructs as a primary outcome were covered in this review. This included: reaction time, error measures, duration of time to complete a task, motor coordination, movement accuracy or the number of successful attempts or trials needed to complete a task [31,32,33,34,35,36,37,38,39]. As for numerical cognition, it can be measured by response time or processing speed [40], accuracy in calculations [41], number sense performance [42], spatial-numerical association measures (e.g., SNARC effect) [43]. We collected data for outcomes whether immediately post-intervention or up after a certain period of time to determine sustainability of the effect of the intervention.

Given the heterogeneity of paradigms and outcome measures, no restrictions were imposed on specific motor effect sizes or task modalities, as the objective of the review was to qualitatively synthesize evidence on number–action interactions rather than to perform a meta-analytic estimation.

### 2.2. Search Methods for Identification of Studies

#### 2.2.1. Search Strategy

Our search strategy included (a) a comprehensive search across various online electronic databases including: Scopus, MEDLINE, PubMed, SPORTDiscus, PsycINFO and SpringerLink; (b) a search of websites and journals, and it included but not limited to IEEE Xplore; (c) a review of citation lists within retrieved articles; and (d) an examination of pertinent conference proceedings, trial records, and research registries, in addition to reaching out to trial investigators, researchers, and experts within our specific research area. To ensure the reproducibility of our search strategy, the exact Boolean logic applied to the Scopus database is detailed below. The search string employed was: (TITLE-ABS-KEY(“Numerical cognition” OR “Number processing” OR “Mental calculation” OR “Arithmetic” OR “Mathematics”) AND TITLE-ABS-KEY(“Motor activity” OR “Motor skills” OR “Movement” OR “Motor control” OR “Motor performance” OR “Kinematics”)) AND NOT TITLE-ABS-KEY(“Child*” OR “Adolescen*” OR “Athlete*”)

This syntax was adapted for other databases (PubMed, MEDLINE, SPORTDiscus, PsycINFO, SpringerLink) using their respective field codes (e.g., MeSH terms for PubMed) and operators to maintain consistency across platforms.

Studies published from 2000 through 26 August 2025, were considered.

#### 2.2.2. Search Strategy Structured According to PICO

The literature search strategy was explicitly informed by the PICO framework to ensure systematic identification and selection of relevant studies. Search terms were grouped according to the Population, Intervention, and Outcome components, while the Comparison component was addressed through experimental contrasts within included studies rather than as a search constraint.

For the Population (P), terms related to human participants were included (e.g., adult, healthy participants). For the Intervention (I), terms referring to numerical cognition and motor control were used, including number processing, numerical magnitude, arithmetic, calculation, and number cognition. For the Outcomes (O), terms related to motor performance were included, such as motor performance, movement, action, posture, kinematics, force, motor control, and sensorimotor.

These groups of terms were combined using Boolean operators (AND/OR) to capture studies investigating interactions between numerical cognition and motor performance. This approach ensured sensitivity to diverse experimental paradigms while maintaining alignment with the research question.

#### 2.2.3. Selection of Studies

**Figure 1:**
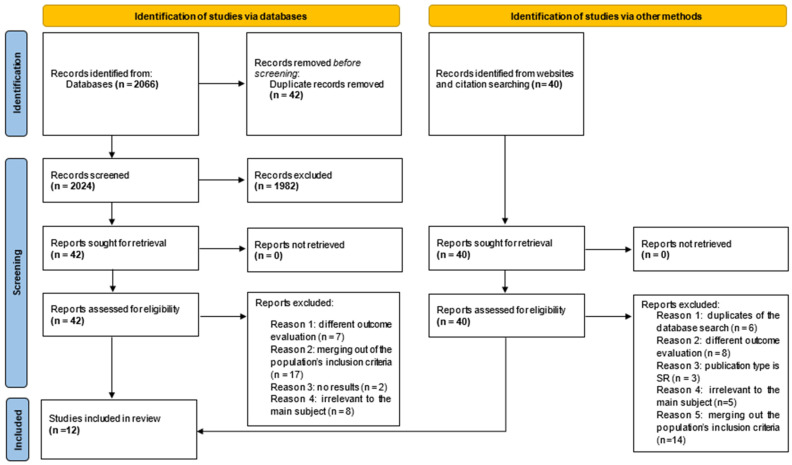
PRISMA flowchart of the database and other sources search.

Following PROSPERO registration under the license CRD420251101463, we adhered to the Preferred Reporting Items for Systematic Reviews and Meta-Analyses (PRISMA) for transparent reporting of relevant studies [44] (see supporting files for PRISMA 2020 checklist). A PRISMA flow diagram briefing our search results was also expanded (Figure 1). Reasons that studies were excluded during full-text screening comprised duplicates between databases and other sources papers, irrelevant or unsuitable interventions with our topic and selection criteria like unhealthy population or participants suffering from dementia, outcome assessment inadequate with our outcome evaluation criteria like psychomotor effects, in addition to articles conducting a study type outside this review’s inclusion selection, and uncompleted studies relying on predictive results.

The retrieved publications were reviewed using the foregoing inclusion and exclusion criteria. Two blinded review authors (ER and AR) participated in the screening separately, to identify articles with potential relevance, by titles and abstracts. We operated on Rayyan (RRID:SCR_017584), a web-based systematic review program [45], for the screening procedure. We then screened for full text and assessed eligibility depending on our preset criteria. Conflicts and disagreements were resolved by consulting a third review author (JA).

#### 2.2.4. Data Extraction and Management

Completing the aforementioned criteria, data from the included articles was extracted and classified into a data abstraction form designed for this review. Collected information was organized in summary tables. Elaborated abstraction table displayed study name including author/s names and year of publication, sample characteristics (e.g., sex, age, condition), study design (e.g., presence of manipulation check, random allocation, random selection), intervention and control description (e.g., protocol, duration), assessed outcomes (e.g., timing, measurement, but not statistical results), funding of the study and conflicts of interest of study authors. Each study was listed per author and organized ascendingly per year of publication. Non-RCTs and RCTs were listed separately in one table (see Appendix A).

We processed data from studies in two categories: (1) investigating the relation between numerical cognition and motor performance, (2) the reverse relationship (the effect of physical movement on numerical cognition). Additionally, data was abstracted by two review authors familiar with the area of cognitive strategies (ER and AR), allowing for a thorough assessment of the sought literature and ensuring that only relevant studies get included in the final analysis step. Data was initially extracted by one author then reviewed by another. Discrepancies have been resolved through discussion.

### 2.3. Risk of Bias Assessment in Included Studies

The Cochrane Risk of Bias 2 (RoB 2.0) assessment tool was used to evaluate the risk of bias amid the randomized studies [46] in its RoB 2.0 Excel Macro Form (Beta Version 7). For Non-randomized studies, (ROBINS-I) tool was adopted for assessing risk of bias [47]. The RoB appraisal was performed autonomously by two authors (ER and AR). When there was no consensus, the author (JA) resolved conflicts. Risk-of-bias plots were created using the Cochrane Robvis visualization tool using datasets.

### 2.4. Role of Task Familiarity/Prior Experience

#### Coding Studies for Task Familiarity and Prior Experience

We classified each included experiment according to the likely role of prior sensorimotor experience in mediating task responses. Two categories were applied: (1) novel/not-overlearned tasks—experimental paradigms that required participants to perform movements or motor responses that were not routine or culturally overlearned or not typically used in daily life (e.g., unconventional finger trajectories, experimentally defined spatial–numeric mappings, atypical movement–number associations, newly instructed whole-body movement mappings), and (2) overlearned/experience-mediated tasks—paradigms in which motor responses relied on highly practiced or habitual sensorimotor routines (e.g., finger movements linked to counting, familiar grasping/reaching, culturally reinforced number–finger associations). Two authors (ER, AR) independently rated studies; disagreements were resolved by consensus. This coding enabled secondary analyses examining whether facilitative or interfering effects were differentially distributed across novel versus overlearned tasks. The rationale and coding criteria followed suggestions in the embodied-numerical cognition literature advocating separation of intrinsic magnitude effects from those driven by learned motor routines [48].

## 3. Results

An initial screening was conducted on 2066 articles retrieved through database searches, complemented by an additional 40 articles identified via manual searches of journals, websites, and citations, using the Rayyan (RRID:SCR_017584) web-based platform. After removing duplicates and excluding studies that did not meet inclusion criteria based on title and abstract evaluations, the selection was narrowed to 42 articles from database searches and 40 articles from alternative sources. These remaining articles underwent full-text review against predefined inclusion criteria, resulting in the final inclusion of 12 studies, which are reported in references as [31,32,33,34,35,36,37,38,39,49,50,51].

### 3.1. Characteristics of Included Studies

The literature analysis provided insight into the study designs and sample characteristics applied by researchers. This enabled the identification of key descriptive elements. A summary of the 12 included studies is presented in Table 1 and Appendix A. Demographic and basic trial information were extracted and compiled in (Table 1), while additional data abstraction details are reported in (Appendix A).

#### 3.1.1. Design

A total of 12 studies met the inclusion criteria for this systematic review. Of these, 2 were randomized controlled trials (RCTs) [39,51], while the remaining 10 employed non-randomized study designs [31,32,33,34,35,36,37,38,49,50].

#### 3.1.2. Year of Publication

With respect to publication year, 10 studies were published between 2010 and 2019 [32,33,34,35,36,37,39,49,50,51], while only one study was published after 2020 [38] and another between 2000 and 2009 [31].

#### 3.1.3. Participants and Sample Size

Regarding participants gender, 2 studies included male-only samples [38,39], while no studies reported female-only samples. The majority of studies (n = 7) involved mixed-gender samples [32,34,35,36,37,49,50], whereas three studies did not specify gender distribution [31,33,51].

Sample sizes showed substantial variability, ranging from fewer than 20 participants in two studies [33,34] to over 100 participants in one study [39]. The most common sample size category was 40–59 participants (n = 5) [31,35,36,50,51], followed by 20–39 participants (n = 1) [37], 60–79 participants (n = 1) [38], 80–99 participants (n = 2) [32,49].

#### 3.1.4. Interventions

We reviewed 12 experimental studies investigating the two-way relationship between motor performance and numerical cognition. Seven studies examined how numerical stimuli, such as digits or number words, influenced motor tasks or recorded motor responses triggered by numbers [31,33,36,37,38,39,49]. Another seven studies focused on the impact of explicit mental calculation or arithmetic tasks on motor performance [32,34,35,38,39,50,51]. In addition, seven studies explored the opposite direction; how motor actions or kinematic manipulations influenced numerical processing [31,32,33,36,37,39,51]. Some studies addressed more than one intervention type, such as combining calculation with motor tasks. All interventions were brief, trial-based manipulations carried out within single experimental sessions, rather than extended training programs.

#### 3.1.5. Controls

Most studies used within-subject designs comparing congruent and incongruent pairings of numerical and motor factors. Four studies compared active calculation or movement conditions against a non-cognitive or immobile baseline [32,34,38,39]. Three studies employed spatial–numerical congruency versus incongruency paradigms to examine compatibility effects [35,36,38]. One study included a control condition using non-numerical symbols [37]. Typically, numerical stimuli; such as small versus large numbers or odd versus even numbers, were presented in a counterbalanced within-subject design [31,33,36,37,49]. Several studies spanned more than one comparator category based on their experimental design.

#### 3.1.6. Outcomes

The majority of studies reported short-term behavioral or kinematic outcomes. Six studies measured reaction times in response to numerical or motor conditions [31,33,38,49,51]. Three studies assessed squat vertical jump performance [32,39,51]. Two studies focused on grip aperture or reach kinematics [31,33]. One study examined muscle activity using surface electromyography and force output [34]. Two studies evaluated the accuracy of arithmetic responses when calculation was the intervention [35,50]. Another study analyzed movement trajectory and velocity in whole-body or kicking actions [37]. None of the studies reported long-term functional, clinical, or patient-reported outcomes.

#### 3.1.7. Sources of Funding

Seven studies did not provide any information about their sources of funding or stated any disclosure about conflict of interest, while 4 studies clearly reported these two points.

### 3.2. Contextual Characteristics of the Included Studies

Beyond the reported experimental results, the twelve selected studies also yielded several contextual insights that illuminate both the methodological evolution of the field and the theoretical justifications underlying each investigation (see Table 1 for study-level details). Overall, the literature remains modest in scope, comprising only twelve empirical reports, and exhibits considerable heterogeneity in design. This variability is evident not only in the diversity of tasks and outcome measures employed but also in the varied contexts in which the studies were conducted.

#### 3.2.1. Geographic and Institutional Distribution of the Evidence

A systematic inspection of the twelve selected studies reveals a marked geographic clustering of research activity. The majority of investigations were carried out by European groups, with Italy and France/Lebanon providing the most substantial contributions, followed by additional European laboratories and comparatively limited representation from Asia and North America. Italian research teams account for a sizable fraction of the evidence, particularly studies addressing numerical tasks, grasping kinematics, and number–action congruency effects [31,33,35,36,37]. These conducts were typically elaborated in labs specializing in embodied cognition, sensorimotor control, and numerical representation, reflecting a strong theoretical focus on grounding numbers within perception–action systems. French laboratories also feature prominently, especially in investigations of mental calculation on complex motor performance such as jumping, force production, and electromyographic responses [32,34,38,39]. Such studies often emerge from institutions with expertise in motor control, biomechanics, and human-movement sciences, offering a complementary, performance-oriented perspective. Outside Europe, only a few studies derive from Asian groups, notably those examining movement–number compatibility through whole-body or composite movements, while North American contributions remain minimal.

Overall, the field appears to be driven primarily by institutionally concentrated programs within interdisciplinary laboratories that bridge cognition, perception, and movement science, with geographic expansion remaining modest.

#### 3.2.2. Motivations and Theoretical Rationales Underpinning the Studies

Across the twelve included investigations, two primary theoretical motivations recur. The first, and most prevalent, derives from embodied cognition and magnitude-processing frameworks such as the Triple Code Model and A Theory of Magnitude (ATOM). Research grounded in this perspective seeks to determine whether numerical magnitude is automatically mapped onto sensorimotor parameters and whether numbers directly influence action planning and execution. This rationale is especially evident in experiments that assess grip-aperture scaling, reach kinematics, and spatial number–movement congruency, where numerical magnitude is expected to modulate motor parameters without explicit strategic intent [31,33,37,49]. These studies typically employ simple or tightly controlled motor outputs to isolate automatic number-action mappings.

A second cluster of work adopts a functional and applied orientation, investigating whether engaging numerical cognition—particularly mental calculation—can modulate motor performance in ecologically relevant tasks. Such investigations examine outcomes including jump height, force production, muscle activation, and movement accuracy, drawing on sport-science and motor-learning frameworks [32,34,38,39,51]. In this view, numbers function as active cognitive drivers that may enhance movement via shared neural resources, arousal mechanisms, or predictive control processes.

A smaller subset explicitly addresses resource-competition, testing whether numerical processing interferes with motor execution under high cognitive load. These studies deliberately increase task complexity to probe the boundary conditions of facilitation, revealing circumstances in which interference may emerge due to task complexity [35,50].

Commonly, the theoretical motivations illustrate a field trajectory that moves from establishing basic number–action correspondences to probing mechanisms, constraints, and applied implications of numerical–motor coupling across diverse motor contexts.

### 3.3. Risk of Bias (RoB) Evaluation

**Figure 2:**
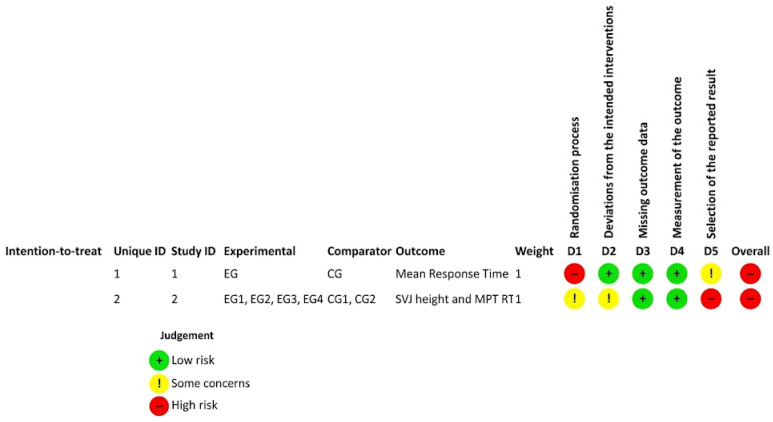
Traffic light plot showing the results of the risk of bias assessment of the randomized studies in our review, ordered according to RCT’s order in data abstraction table. Risk of Bias. Domains: D1—Randomization process; D2—Deviations from Intended Interventions; D3—Missing outcome Data; D4—Measurement of Outcomes; D5—Selection of the Reported Result.

**Figure 3:**
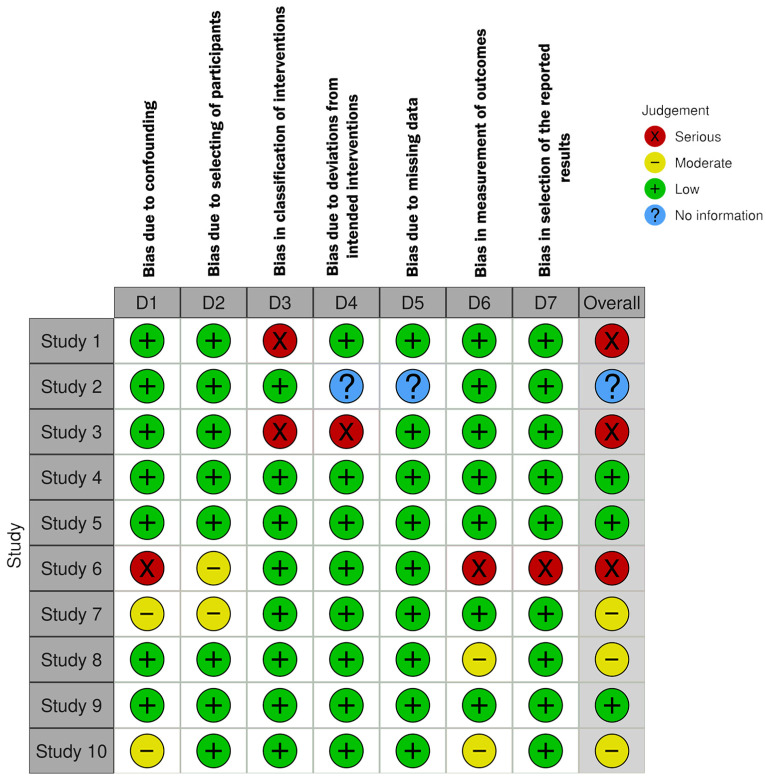
Traffic light plot showing the results of the risk of bias assessment of the non-randomized studies in our review, ordered according to non-RCTs in data abstraction table. Risk of Bias. Domains: D1—Bias due to Confounding; D2—Bias in Selection of Participants; D3—Bias in classification of interventions; D4—Bias due to Deviations from Intended Interventions; D5—Bias due to Missing Data; D6—Bias in Measurement of Outcomes; D7—Bias in Selection of the Reported Result.

**Figure 4:**
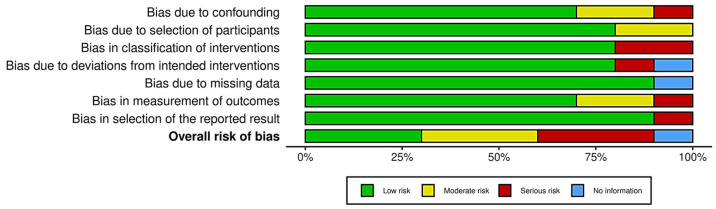
Graphic showing the results of the risk of bias assessment of the non-randomized studies in our review, separated by specific Cochrane Risk of Bias domain.

The detailed risk of bias assessment results are shown in Figure 2, Figure 3 and Figure 4.

#### 3.3.1. RoB in RCTs

Both randomized controlled trials were evaluated using the RoB 2 assessment tool. The 2 RCTs raised some concerns about bias but were not considered at high risk across all domains. In the study of (Bal, 2018) [51] referred as study 1 in the traffic plot in Figure 2, the randomization process lacked clear description, leading to concerns about allocation sequence generation and concealment. Participant blinding was not possible due to the intervention, but outcome measures: reaction times and jump performance, were objectively recorded, minimizing measurement bias. There was no indication of missing outcome data or selective reporting.

In the study of (Khayat, 2019) [39] referred as study 2 in the traffic plot in Figure 2, the randomization method was described but lacked sufficient detail on allocation concealment, causing some concerns. Participant blinding was absent, which could allow deviations from intended interventions; however, outcomes such as jump height and reaction times were objective, reducing the risk of assessor bias. No missing data or selective reporting were detected.

Overall, both RCTs presented outcome data judged at low risk or with some concerns in most bias domains. Still, unclear randomization and allocation concealment procedures limited the certainty of the evidence (Figure 2).

#### 3.3.2. RoB in Non-RCTs

Most included studies were non-randomized controlled trials or within-subject designs, assessed using the ROBINS-I tool [31,32,33,34,35,36,37,38,49,50]. Study number from 1 to 10 as seen in the traffic light plot of Figure 4 was designated according to the order of non-RCTs in Appendix A.

Overall, the risk of bias in these non-RCTs ranged from moderate to serious, especially in the domains of confounding and participant selection. Most studies relied on convenience samples of undergraduate students, often without clear justification or control for confounders, increasing the risk of selection bias.

Deviations from intended interventions were common because blinding was not applied, and participants were aware of the experimental conditions. However, since outcomes were mostly objective (e.g., reaction times, motor performance), measurement bias was generally low.

Missing data rarely posed a problem, as most studies reported complete or nearly complete outcome data. Selective reporting was harder to evaluate due to a lack of pre-registered protocols, resulting in some concerns about reporting bias.

In summary, non-randomized studies were mostly judged to have moderate risk of bias, with several studies showing serious risk related to confounding and limited methodological safeguards [31,32,38] (Figure 3 and Figure 4).

### 3.4. Effects of Intervention

#### 3.4.1. Numerical Stimuli and Motor Performance

Seven studies explored how numerical stimuli such as digits, number words, or magnitudes, affect motor task execution [31,33,36,37,38,39,49]. Consistently, these studies found that when numerical information aligned with the direction or scale of movement, motor responses were facilitated. For instance, grip aperture and reach trajectories varied systematically with number size, with smaller numbers triggering smaller grip openings [31,33]. Reaction times also improved when small numbers were paired with leftward or downward movements, and large numbers with rightward or upward actions [36,38,49]. The study of Rugani (2018) provided compelling evidence that this effect was specific to symbolic numerical stimuli, as non-symbolic symbols did not influence motor kinematics, indicating a number-specific modulation of motor execution [37].

#### 3.4.2. Mental Calculation and Motor Performance

Another set of seven studies examined the impact of mental calculation on subsequent motor performance [32,34,35,39,50,51]. The majority (n = 5) reported facilitative effects: mental arithmetic was associated with increased squat vertical jump heights [32,38,39,51] and enhanced force output and electromyographic activity during muscle contractions [34]. These findings suggest that engaging numerical processing may elevate arousal or motor drive, thereby boosting motor performance. Conversely, two studies observed interference effects, including slower responses under cognitive load [35] and diminished movement accuracy and speed [50]. Despite these exceptions, the overall evidence predominantly supports a positive influence of numerical cognition on motor function.

#### 3.4.3. Motor Actions Influencing Numerical Cognition

Seven studies also investigated how motor actions or kinematic manipulations affect numerical cognition [31,32,33,36,37,39,51]. Across these studies, motor activity consistently biased numerical processing in a congruent and facilitative manner. Specifically, movements directed leftward or downward facilitated processing of smaller numbers, whereas rightward or upward movements enhanced processing of larger numbers [36,39]. Additionally, grasping and reaching movements influenced both motor parameters and subsequent numerical judgments [31,33]. Whole-body actions such as jumping, turning, or kicking similarly modulated numerical estimates in line with spatial–numerical associations [32,37,51]. These convergent findings underscore the role of motor performance in shaping numerical cognition.

#### 3.4.4. Influence of Task Familiarity and Prior Experience

To investigate whether number–action effects are influenced by prior sensorimotor experience, the reviewed studies were categorized based on task familiarity. Among the twelve empirical studies examined, seven utilized overlearned motor responses, predominantly involving habitual finger movements or familiar reaching and grasping actions [31,32,33,34,35,49,50]. Conversely, five studies employed novel or less practiced motor mappings, requiring participants to perform experimentally defined or non-habitual movement–number associations [36,37,38,39,51]. Of particular note, Rugani et al. (2018) implemented a paradigm specifically designed to reduce reliance on habitual finger-counting strategies by dissociating numerical magnitude from conventional motor routines, thus offering a rigorous test of magnitude–action coupling in a novel task setting [37]. These findings align with previous work by the same group (Rugani et al., 2017), which demonstrated that numerical magnitude systematically influenced both action selection and finger movement kinematics even when movements were unfamiliar within numerical contexts [48].

Utterly, these results provide convergent evidence that numerical magnitude can shape motor planning processes independently of well-established sensorimotor associations. While facilitative interactions between numerical magnitude and motor performance were observed across both categories, the effects observed in novel task paradigms offer particularly strong support for a direct influence of numerical magnitude on motor planning and execution beyond the contribution of ingrained sensorimotor routines.

#### 3.4.5. Summary of Effects and Reciprocal Relationship

Among the 12 studies reviewed, 10 reported facilitative interactions between numerical cognition and motor performance, while 2 documented interferences. All seven studies presenting numerical stimuli during motor tasks demonstrated facilitatory congruence effects. With mental calculation concurrent to motor tasks, five studies found enhancement of motor outcomes, while two reported interferences. Similarly, all seven studies examining motor actions’ effects on numerical cognition showed facilitative congruence effects. A summary of findings on effect direction in the included studies is presented in (Table 2) of this review.

## 4. Discussion

### 4.1. Synthesis of Findings by Behavioral Mechanism

The aim of the present systematic review was to investigate the mutual relationship between numerical cognition, more specifically, the number and/or arithmetic processing, and motor performance, such motor activity. This review integrates findings from 12 experimental studies that explored the bidirectional relation between numerical cognition and motor performance. The prevailing tendency across these investigations was facilitative. Rather than reflecting a single unitary process, the twelve included studies converge on two primary behavioral mechanisms through which numerical cognition and motor performance interact. These mechanisms differ in the nature of the motor outcome and the role played by numerical processing, allowing a more structured interpretation of the literature.

#### 4.1.1. Spatial–Numerical and Kinematic Associations

The first mechanism involves spatial–numerical associations, whereby numerical magnitude systematically modulates motor kinematics and spatial parameters. Five studies demonstrated that processing numerical magnitude influenced movement direction, grip aperture, reach trajectory, or postural adjustments, such that smaller numbers facilitated leftward or precision-oriented movements, whereas larger numbers facilitated rightward or power-oriented movements [31,33,36,37,49]. These effects were typically observed in tasks requiring rapid motor planning or online control and align with the notion that numerical magnitude is automatically mapped onto spatial and motor representations. Importantly, these studies relied on precise kinematic or spatial metrics rather than global performance indices, suggesting that numerical magnitude influences motor planning stages more than movement outcome itself.

#### 4.1.2. Psychophysiological Facilitation of Motor Output

A second, distinct mechanism is characterized by psychophysiological facilitation, in which engagement in numerical or arithmetic processing enhances gross motor output. Five studies reported improved motor performance—such as increased vertical jump height, force production, or electromyographic activity—following mental calculation tasks [32,34,38,39,51]. Unlike spatial–numerical associations, these effects were not direction-specific and were observed in whole-body or strength-based movements. The predominance of facilitation over interference suggests that, under sequential or moderate cognitive load conditions, numerical processing may increase motor drive or readiness rather than compete for shared resources. Only two studies reported interference effects, primarily under conditions of increased task complexity [35,50], indicating that facilitation is the dominant pattern within the reviewed literature.

### 4.2. The Role of Task Familiarity and Novelty

An important methodological dimension emerging from the included studies concerns the familiarity of the motor task used to investigate number–action interactions. Several studies relied on overlearned actions, such as habitual reaching, grasping, or culturally ingrained finger-based spatial mappings, where sensorimotor associations may already be well established [31,33,35,49]. In these paradigms, numerical modulation of movement may partly reflect the activation of pre-existing motor routines.

Crucially, however, facilitative number–motor effects were also observed in studies employing novel or experimentally defined mappings, in which participants had no prior experience linking specific numerical values to the required motor output [32,34,37,38,39]. The persistence of facilitation under these conditions suggests that number–action interactions cannot be attributed solely to learned associations or cultural conventions. Instead, they likely reflect a more general functional coupling between numerical processing and motor control that operates even in unfamiliar task contexts.

### 4.3. Theoretical Implications

While the included studies were behavioral, these findings align with neuroimaging literature suggesting the involvement of a complex, integrative network extending beyond isolated cortical areas, involving dynamic interchange among multiple brain regions and circuits.

Despite the fact that the present systematic review focused exclusively on behavioral outcomes, the observed patterns of interaction between numerical cognition and motor performance can be discussed in relation to existing neurocognitive literature. Prior neuroimaging research has consistently implicated parietal and motor-related regions in numerical processing, particularly the intraparietal sulcus (IPS), which plays a central role in numerical magnitude representation and also contributes to spatial attention and sensorimotor integration [4,5]. Furthermore, functional connectivity between the IPS and premotor or supplementary motor areas has been reported during tasks involving spatial coding and movement preparation in numerical contexts [21,52].

However, it is essential to emphasize that none of the studies included in this systematic review directly assessed neural activity or neurophysiological mechanisms. Consequently, references to distributed parieto-frontal networks or subcortical structures, such as the cerebellum and basal ganglia, should be regarded as interpretative and hypothesis-generating, based on established neurocognitive models rather than on evidence derived from the reviewed studies themselves. Accordingly, the present findings do not permit specific claims regarding neural circuitry, but instead highlight the need for future research combining behavioral paradigms with neuroimaging or neurophysiological methods to directly examine the neural substrates underlying numerical–motor interactions.

Finally, embodied cognition accounts gain strong support from this synthesis, affirming that rather than being processed in isolation, numbers are dynamically grounded in sensorimotor systems, as evidenced by the bidirectional and context-general effects observed [18,19]. Importantly, the current review extends prior claims by demonstrating that motor actions not only reflect but actively shape numerical judgments, emerging a bidirectional embodied loop between numerical cognition and motor behavior.

### 4.4. Influence of Task Familiarity on Number–Motor Interactions

Synthesizing across studies, task familiarity emerges as a relevant moderating factor in number–motor interactions. Seven studies employed overlearned motor actions, such as habitual reaching or grasping, where numerical modulation may capitalize on existing sensorimotor associations [31,33,35,36,37,49,50]. However, importantly, the remaining five studies that introduced novel or less-practiced motor mappings also demonstrated facilitative numerical effects, particularly in force production and whole-body movements [32,34,38,39,51]. This convergence across familiar and novel tasks strengthens the interpretation that numerical cognition can modulate motor performance beyond simple experience-based learning, supporting the existence of a functional interaction that generalizes across different levels of task familiarity.

### 4.5. Strengths and Limitations of the Evidence

Based on the available literature, this is the first systematic review to integrate findings on the reciprocal relationship between numerical and motor domains within a cognitive neuroscience perspective. While prior research has investigated facets of this interplay, this synthesis demonstrates consistent facilitation across diverse paradigms and participant groups, bridging previously fragmented literatures in psychology, motor control, and sport science in order to establish a consolidated foundation for understanding how numerical and motor processes interact.

However, this review addresses a critical gap in the literature. Although motor-cognitive interactions have been extensively studied in contexts like dual-task gait and executive-motor coupling, the specific relationship between numerical cognition and motor action has received comparatively little systematic attention. This gap is consequential, not only for advancing theoretical models but also for its translational potential. The findings indicate promising paths for designing interventions in educational settings, athletic training, and neurorehabilitation, where numerical and motor skills frequently converge.

The evidence base included in this review demonstrates several strengths. Objective and sensitive performance measures such as reaction times, kinematic trajectories, force output, and electromyography, were consistently employed, enhancing the reliability of observed facilitative effects across different tasks and modalities. Nevertheless, certain limitations must be acknowledged. One is the insufficiency of direct neural evidence in the included studies. Most relied exclusively on motor-behavioral outcomes such as reaction times, kinematics, or EMG, with little incorporation of neuroimaging, electrophysiological, or neurostimulation techniques. This gap limits mechanistic interpretations and emphasizes the need for future work explicitly targeting brain substrates, especially during arithmetic. Moreover, from methodological standpoint, only two studies employed randomized controlled designs, and both exhibited methodological concerns related to randomization and allocation concealment. The majority of studies were non-randomized and often involved small convenience samples, primarily comprising students. None reported blinding procedures, although the use of objective outcome measures likely mitigated measurement bias. Additionally, no studies examined long-term or functional outcomes, restricting conclusions about the persistence and ecological validity and durability of these effects.

In summary, while the current evidence supports an emerging and reciprocal link between numerical cognition and motor performance, future research should aim to strengthen methodological precision, expand sample diversity, and investigate the durability and practical relevance of these interactions.

### 4.6. Implications for Practice and Research

These reciprocal interactions carry significant implications for both practice and future research. In educational contexts, incorporating motor activities into numerical learning may develop on natural embodied mappings to enhance numerical skill development in children. Within sport science, integrating numerical tasks into training regimens could potentially boost motor performance by exploiting the facilitative influence of cognitive engagement. Similarly, in rehabilitation settings, interventions targeting both motor and numerical cognition might offer benefits for patients with neurological impairments, by addressing intertwined cognitive-motor deficits.

Future investigations should prioritize moving beyond brief, laboratory-based experiments to evaluate the effects of longer-term, environmentally valid interventions with clinical and functional outcomes. Rigorous randomized controlled trials are essential to confirm the strength of the observed effects, explore moderating factors like task complexity or individual differences, and assess the durability of intervention benefits. Critically, more research should incorporate neuroimaging and neurophysiology to directly test whether behavioral facilitation corresponds to structural or functional changes in fronto-parietal, cerebellar, and basal ganglia circuits. Such work would not only refine theoretical models like the TCM and ATOM but also clarify the mechanisms through which cognition and action mutually reinforce one another.

### 4.7. Limitations and Certainty of Evidence

In response to the heterogeneity of the included studies, we applied the principles of the GRADE framework to assess the overall certainty of the evidence [53]. Given that the majority of included studies (10 out of 12) utilized non-randomized designs, the baseline certainty was initially graded as Low. This assessment was further impacted by the risk of bias analysis, which identified moderate to serious concerns, particularly in the domains of confounding and participant selection (see Section 3.3.2). Additionally, imprecision was a limiting factor due to small sample sizes in several studies. Consequently, the overall certainty of the evidence regarding the bidirectional interaction between numerical cognition and motor performance is rated as Very Low. This indicates that while the current body of literature consistently points toward a facilitative relationship, the estimated magnitude of this effect is uncertain, and future high-quality randomized trials are likely to have an important impact on our confidence in the estimate.

## 5. Conclusions

This systematic review suggests that numerical cognition and motor performance are functionally coupled rather than isolated abilities. The synthesized evidence indicates a prevailing trend of facilitation, where numerical processing appears to modulate motor execution, and motor activity reciprocally influences numerical judgments. However, these findings should be interpreted with caution given the limited number of studies and the methodological heterogeneity observed. While the current evidence is strictly behavioral, the observed interactions align with theoretical frameworks proposing shared neural resources within fronto-parietal, premotor, and cerebellar networks. By illuminating the overlapping relationship between number and action, this review advances the theoretical understanding of cognitive-motor integration and highlights promising potential avenues for translational applications in education, sport, and rehabilitation.

## Figures and Tables

**Table 1:** Descriptive and Demographic Data of Included Studies.

	Number of Studies
Total number of studies	12
Type of study	
RCT	2
non-RCT	10
Type of intervention	
Numerical cognition strategy	8
Physical activity/motor action	4
Population gender	
Male only	2
Female only	0
Combined	7
Not stated	3
Sample size	
<20	2
20–39	1
40–59	5
60–79	1
80–99	2
100+	1
Decade of study published	
2020–onwards	1
2010–2019	10
2000–2009	1

**Table 2:** Summary of Findings on intervention’s Effect Direction in Included Studies.

Comparison (Intervention vs. Control)	Outcomes Assessed	Number of Studies (with References)	Direction of Effect
Numerical stimuli (digits, magnitudes) prior to motor tasks vs. control/baseline	Reaction times, reach/grasp kinematics, movement trajectories	7 studies [31,33,36,37,38,39,49]	All studies showed facilitation of motor responses by numerical stimuli (faster RTs, altered kinematics in congruent conditions).
Mental calculation/arithmetic tasks prior to motor performance vs. baseline	Jump height, EMG, force, motor accuracy	7 studies [32,34,35,38,39,50,51]	Positive/facilitation: [32,34,38,39,51]. Negative/interference: [35,50]. Overall: predominantly facilitation.
Motor actions (reaching, grasping, jumping, turning) influencing numerical cognition	Arithmetic accuracy, response times to numbers, number-space compatibility	7 studies [31,32,33,36,37,39,51]	All studies showed that motor actions biased numerical cognition: congruent movements improved performance, incongruent impaired it.
Symbolic vs. non-symbolic control conditions	RTs and movement kinematics	1 study [37]	Only numerical symbols modulated motor performance; non-symbolic controls had no effect.
Congruent vs. incongruent number–movement pairings	RTs, calculation accuracy	3 studies [35,36,38]	Congruent pairings facilitated responses; incongruent pairings slowed or reduced accuracy.
Motor imagery vs. physical execution	Jump height, timing	1 study [32]	Mental calculation facilitated both imagery and execution, with weaker effects in imagery.

## Data Availability

All data about the systematic review is available on PROSPERO.

## Abbreviations

The following abbreviations are used in this manuscript:

IPS	Intraparietal Sulcus
TCM	Triple Code Model
ATOM	A Theory of Magnitude
RCT	Randomized Controlled Trial
PRISMA	Preferred Reporting Items for Systematic Reviews and Meta-Analyses
RRID	Research Resource Identifier
RoB	Risk of Bias
RoB 2.0	Cochrane Risk of Bias 2.0 Tool
ROBINS-I	Risk Of Bias In Non-randomized Studies—of Interventions
EMG	Electromyography
SNARC	Spatial–Numerical Association of Response Codes

## Appendix A

**Table A1:** Characteristics of Included Studies.

Study Name	Study Design	Participants Description	Intervention Description	Control Description	Outcomes Assessed	Funding of the Study	Conflicts of Interest of Study Authors
Non-Randomized Controlled Trials							
O. Lindemann et al., 2007 [31]	Non-RCT Trial (within-subjects design)	Exp.1: N = 14 novice healthy students. Exp.2: N = 22 novice healthy students. Exp.3: N = 18 novice healthy students.	Exp.1: reach out and grasp the object with a power or a precision grip depending on the parity of an Arabic digit number. Exp.2: same as Exp.1 but instead of grasping, MPT was required either to the small top or to the large bottom segment of the object. Exp.3: same as Exp.1 but grasping only with thumb and index fingers.	_	Reaction times for different grips. Maximum grip aperture.	Not provided.	Not provided
T. Rabahi et al., 2012 [32]	Non-RCT Trial	Exp.1: N = 43, 28 men (21.5 ± 2.6 y.o.) and 15 women (22.1 ± 1.6 y.o.). Exp.2: N = 30, 16 men (23.7 ± 2.5 y.o.) and 14 women (22.1 ± 2.2 y.o.). Exp.3: reference participants, N = 10 men (22.1 ± 2.4 y.o.). All novice, students.	Participants performed SVJ after cognitive tasks like specific action verb pronunciation(or silent AV), mental calculation (MS), and kinesthetic imagery.	Same experimental procedure as intervention groups but without performing the cognitive tasks.	Height of SVJ.	Not provided.	No conflicts of interest reported.
R. Chiou et al., 2012 [33]	Non-RCT Trial (within-subjects design)	N = 13 undergraduates, right-handed, healthy, naive to the purpose of the experiment, experienced in using Arabic digits.	Participants performed visual open-loop grasping towards objects with Arabic digits labels. They had to grasp the object labelled with the odd number if they heard a tone of the higher frequency, whereas they had to grasp the object labelled with an even number if they heard a lower tone frequency.	_	Evolution of grip aperture relatively to numerical magnitude with different RT and MT evaluated.	Not provided.	Not provided.
L. Bensoussan et al., 2012 [34]	Non-RCT Trial (within-subjects design)	N = 10 healthy subjects. 8 right-handed and 2 left-handed. 4 women and 6 men aged 20 to 35 y.o.	Subjects performed wrist isometric extension while receiving mental arithmetic task conditions (subtraction counting backwards in seven-aloud and silent).The period of counting lasted for 10 s and was delimited by two auditory signals.	No MA task was requested during the 10 s elapsing between both signals. The design of the trials was exactly the same in control conditions than in MA conditions.	Mean inter-spike interval (ISI), ISI variability, surface EMG activity, force level, activation of wrist extensor SMU.	Financial support from CNRS.	Not provided.
M. Hartmann et al., 2012 [49]	Non-RCT Trial (within-subjects design)	Exp.1: N = 24, 21 female, 3 male, all right-handed. Age ranging from 19 to 35 y.o. Exp.2: N = 24, 4 male, 20 female, all right-handed. Age ranging from 20 to 35 y.o. Exp.3: N = 36 subjects (8 male, all right-handed). Age ranging from 20 to 28 y.o. All are undergraduate students.	Participants positioned on a motion platform. Exp.1: random-number generation task while blindfolded and moving passively in various directions and at stationary. Exp.2: asked to decide as fast as possible whether an auditorily presented number was smaller or larger than 5 Exp.3: asked to decide as fast as possible whether they were moved leftward or rightward. In each trial, either a small (“one, two”) or a large (“eight, nine”) number was presented.	_	Magnitude of generated numbers. Reaction time (RT) of response on buttons held by subjects.	Swiss National Science Foundation.	Not provided.
L. Lugli et al., 2013 [35]	Non-RCT Trial (within-subjects design)	Exp.1: N = 56 healthy university students (30 females, mean age = 22 y.o.). Exp.2: N = 30 new students (16 females, mean age = 20.4 y.o.) from the same pool.	Exp.1: Participants were asked to perform additions or subtractions with ascending or descending movements (Passive (elevator) and active (stairs) modes) for 22 s and to say the result of each calculation aloud. Exp.2: same as in Exp.1. The only difference was that participants were asked to make the calculations while imagining to perform an upward or downward motion.	_	Congruency between calculation type and movement direction.	MIUR and the European Community, FP7 project ROSSI, Emergence of Communication in Robots through Sensorimotor and Social Interaction.	The authors have declared that no competing interests exist.
F. Anelli et al., 2014 [50]	Non-RCT Trial (within-subjects design)	N = 48 undergraduate students (33 female, mean age: 20.3 y.o., SD 3.3).	Participants were asked to perform additions or subtractions for 22 s and to say the result of each calculation aloud while walking leftward or rightward upon the experimenter’s instructions.	_	Number of correct calculations made during additions and subtractions.	Ricerca Fondamentale Orientata (RFO) funds, University of Bologna, and by the European Community, in FP7 project ROSSI: Emergence of Communication in Robots through Sensorimotor and Social Interaction.	The authors declare that the research was conducted in the absence of any commercial or financial relationships that could be construed as a potential conflict of interest.
X. Cheng et al., 2015 [36]	Non-RCT Trial (within-subjects design)	Exp.1: N = 64 healthy, right-handed subjects (21 males and 43 females), 21.7 y.o. on average (range: 18–28). Exp.2: N = 30 healthy, right-handed subjects (9 males and 21 females), 23.3 y.o. on average (range: 18–28). All participants were naive to the purpose of the experiment.	Participants were required to report numbers between 1 and 30 as randomly as possible with their eyes covered by a mask in three blocks, i.e., head turns, arm turns and a baseline condition. In Exp.1: Lateral head and arm turns. In Exp.2: Composite body movements with congruent and incongruent directions.	_	Impact of spatial processing on single numbers representation. Motion-numerical compatibility effect observed for head and arm movements.	Not provided.	The authors declare that the research was conducted in the absence of any commercial or financial relationships that could be construed as a potential conflict of interest.
R. Rugani et al., 2018 [37]	Non-RCT Trial	N = 23 healthy right-handed students (10 males and 13 females, mean age = 22.74 y.o., SD = 0.75).	Participants sat in front of a monitor, wearing with their right index a small soccer shoe. They were instructed to kick a small ball with their right index towards a soccer goal after observing numbers (A small digit (2), an intermediate digit (5; no-go signal), a large digit (8), and a non-numerical symbol ($)). They refrained from kicking when number five was presented.	_	Trajectory path efficiency, velocity peak, contact time, time of maximum velocity, time to maximum trajectory height.	SIR grant (Scientific Independence of Young Researchers and “German Academic Exchange Service or DAAD” Funding program: Research Stays for University Academics and Scientists, 2017.	The authors declare that the research was conducted in the absence of any commercial or financial relationships that could be construed as a potential conflict of interest.
J. Khayat et al., 2021 [38]	Non-RCT Trial	N = 65 healthy undergraduate male students and had normal or corrected to normal vision.	Warm-up session then subjects (CG and EGs) performed three series of six MPMs with 3 mn rest between two consecutive blocks. EGs 1 to 3 realized the first three MPMs of each block remaining immobile in front of a black wall, as in the CG as a baseline. Then they performed three MPMs each after a cognitive task involving mental calculation and number comparison.	CG remained immobile 10 s in front of a black screen after warm-up and training series, and then executed the MPM task.	Response time (RT) of a manual-pointing movement (MPM).	Not provided.	Not provided.
Randomized Controlled Trials							
O. Bal et al., 2018 [51]	RCT (Pre-post tests)	N = 40 undergraduate healthy Sport Science students aged 19–22 years.	Subjects performed 2 series of 5 mental subtractions (respectively as a Pre-T and as a Post-T). The subjects were instructed to find the correct result of each subtraction as fast as possible. N = 25 subjects performed 5 SVJs with 1 mn rest between 2 consecutive jumps.	Same procedure as EG but after arithmetic series, N = 15 subjects remained seated for 5 min, no physical activity.	Mean of response time (LnRT) of correct responses in mental subtractions before and after performing squat vertical jumps (SVJs).	Not provided.	Not provided.
J. Khayat et al., 2019 [39]	RCT	N = 161 healthy undergraduate male students, mean age = 20 y.o., normal BMI, English-speaking.	Subjects were asked to achieve the highest possible SVJ or MPT reaching movement as fast as possible. After warm-up each subject performed three series of either six SVJs, or six MPTs. In each series the first 3 movements were performed without any previous cognitive task (subjects in front of a black screen during 10 s) as a baseline. The three ensuing movements were performed after a cognitive task, i.e., mental subtraction or after reading loudly a number (during 10 s) or an AV.	No cognitive stimulus was given (subjects simply stood in front of a black screen).	SVJ height (cm) and MPT RT (ms).	Not provided.	Not provided.
